# Early detection of rheumatoid arthritis through patient empowerment by tailored digital monitoring and education: a feasibility study

**DOI:** 10.1007/s00296-025-05793-8

**Published:** 2025-02-04

**Authors:** Nicola Pfeuffer, Fabian Hartmann, Manuel Grahammer, David Simon, Louis Schuster, Sebastian Kuhn, Gerhard Krönke, Georg Schett, Johannes Knitza, Arnd Kleyer

**Affiliations:** 1 Department of Internal Medicine 3, Rheumatology and Immunology, Friedrich-Alexander Universität Erlangen-Nürnberg, Universitätsklinikum Erlangen, Erlangen, Germany; 2 Deutsches Zentrum Immuntherapie, Universitätsklinikum Erlangen and Friedrich-Alexander Universität Erlangen-Nürnberg, Erlangen, Germany; 3 ABATON GmbH, ABATON, Berlin, Germany; 4 Center for Health Services Research, Faculty of Health Siences Brandenburg and Brandenburg Medical School Theodor Fontane, Rüdersdorf bei Berlin, Berlin, Germany; 5 Medizinische Klinik mit Schwerpunkt Rheumatologie und Klinische Immunologie, Charité– Universitätsmedizin Berlin, Corporate Member of Freie Universität Berlin and Humboldt-Universität zu Berlin, Charitéplatz 1, 10117 Berlin, Germany; 6 Institute for Digital Medicine, University Hospital Giessen-Marburg, Philipps University, Baldingstrasse, Marburg, Germany; 7 Université Grenoble Alpes, Grenoble, France

**Keywords:** Digital health, Patient education, Remote monitoring, Rheumatoid arthritis

## Abstract

Patients at risk for rheumatoid arthritis (RA) describe fluctuating and nonspecific symptoms, making it difficult to quantify symptom burden and recognize RA progression. This study aimed to assess feasibility and diagnostic value of a multimodal digital self-monitoring program in preclinical RA. This prospective cohort study included individuals at-risk for RA, who first watched self-produced educational videos about (preclinical) RA and joint self-examination techniques and then started the REMOTRA symptom monitoring. Key outcomes measured included patient acceptance (Net Promoter Score: NPS), monitoring program usability (System usability scale: SUS), monitoring adherence, diagnostic accuracy, and reported symptom burden. A total of 43 participants (65.9% female, mean age 50.1 years) were enrolled. The educational and self-examination videos received NPS ratings of 54.4 and 31.6, respectively. The monitoring software received usability scores of 88.1/100 (SD: 5.5) at three months and 85.4/100 (SD: 16.0) at 6 months. 24/41 (58.5%) completed all questionnaires, and the average app usage was 4.8 months (SD: 1.8). None of the patients with a REMOTRA score below 10 developed RA, yielding a negative predictive value and sensitivity of 100%. However, the positive predictive value was 12%, and the specificity was 42.1%. Analgesic and cortisone usage was reported by 58.5% and 29.3% of participants, respectively. The strong patient acceptance, ease of use, and high adherence rates, combined with encouraging diagnostic outcomes, underscore the potential of this personalized digital monitoring and education approach. These findings suggest that further validation through multicenter studies is warranted.

**Supplementary Information:**

The online version contains supplementary material available at 10.1007/s00296-025-05793-8.

## Background

Rheumatoid Arthritis (RA) is a chronic, inflammatory autoimmune disorder, characterized by fluctuating disease activity and nonspecific symptoms such as morning stiffness and joint pain [1]. The presence of autoantibodies, including anti-citrullinated peptide antibodies (ACPA) and rheumatoid factor (RF), can precede the clinical onset of RA by several year [2]. Individuals who exhibit symptoms alongside these autoantibodies face a markedly elevated risk of progressing to RA [3, 4]. The concept of a “window of opportunity” has been established for RA, emphasizing the importance of timely treatment initiation to mitigate or even halt disease progression [5, 6]. Close monitoring of these high-risk individuals is crucial to ensure timely detection of disease onset [6]. However, close monitoring of at-risk individuals is increasingly constrained by the widening care gap in rheumatology [7]. This gap is exacerbated by a concerning decline in the number of practicing rheumatologists, coupled with a growing influx of both new referrals and established rheumatic patients [7]. Current follow-up strategies typically involve infrequent, pre-scheduled short-duration in-person visits. This approach often fails to detect disease flares occurring between appointments [8]. Moreover, these visits can be burdensome for individuals and frequently seem unnecessary when patients are symptom-free [9]. Additionally, the lack of empowerment, guidance, and certainty exacerbates psychological strain among at-risk individuals [10].

Remote monitoring, coupled with standardized asynchronous digital patient education, has the potential to empower individuals at risk for RA [9, 11], alleviate healthcare constraints [12], and ensure the timely initiation of therapy [13]. Moreover, remote monitoring could minimize unnecessary in-person visits, thereby reducing the burden on patients and freeing up the limited capacity of rheumatology services [8, 12, 14]. Furthermore, this data can be used to generate prediction models, to identify patients with the highest risk of progressing to RA [15] and guiding the selection of the most suitable therapies, thus setting the foundation for precision medicine [16]. The COVID-19 pandemic has accelerated the adoption of remote monitoring within rheumatology [17]; however, no study to date has explored the potential of remote monitoring coupled with digital education in patients at risk for RA.

The REMOTRA (REMote moniToring in pReclinical Arthritis) trial aimed to evaluate the feasibility of this multimodal digital intervention, focusing on patient acceptance, adherence, and diagnostic effectiveness.

## Methods

**Fig. 1:**
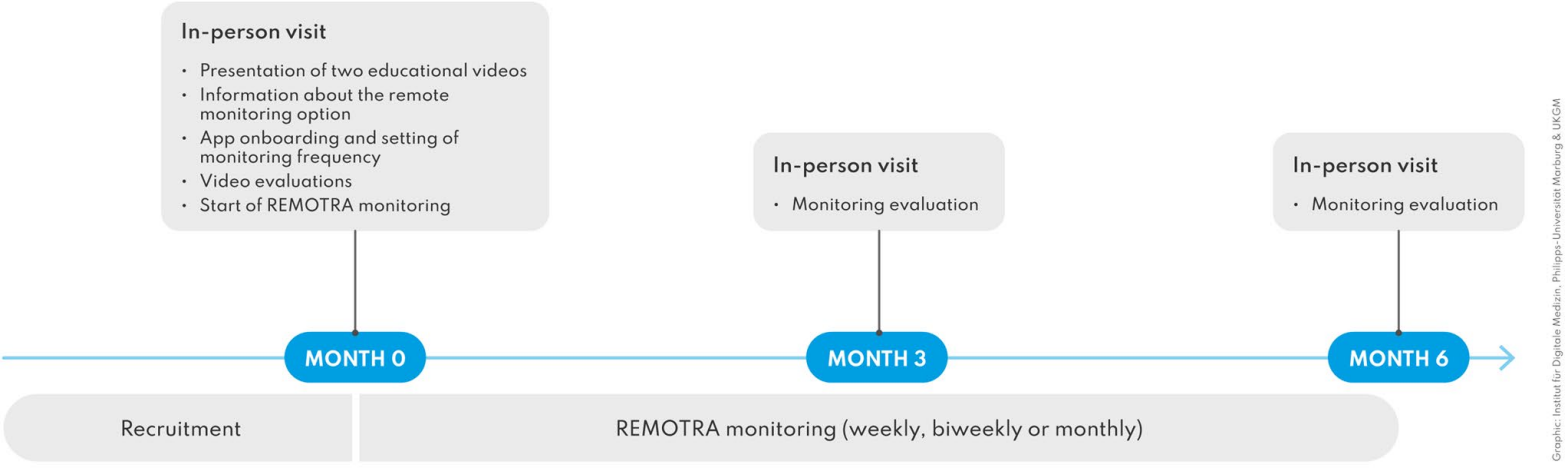
Study design overview

REMOTRA (REMote moniToring in pReclinical Arthritis) was a prospective, open label, 6-months non interventional feasibility study (Fig. 1). The study included regular in-person visits at month 3 and 6 with in-between remote monitoring. The trial was approved by the local ethics authorities (Reg no. 333_16B and 21_21B). All participants provided written informed consent. Figure 1 displays an overview of the study design. Results were analysed and presented according to the CONSORT guidelines for feasibility trials [18]. Interim results were presented at the 2023 EULAR congress as a poster presentation [19].

### Participants

Individuals at risk for RA were recruited from the existing cohort IRACE (Individuals of Risk Arthritis Cohort Erlangen) of the University Hospital Erlangen Patients had to fulfil the following inclusion criteria: Presence ACPA and/or RF, smartphone ownership and no clinically detectable synovial swelling at the baseline visit, thus these individuals did not fulfil the classification criteria for RA. IRACE individuals were informed about the opportunity to participate in the study during regular appointments and through phone calls. The reasons provided by those who declined to participate were recorded.

### Multimodal digital self-monitoring program

**Fig. 2:**
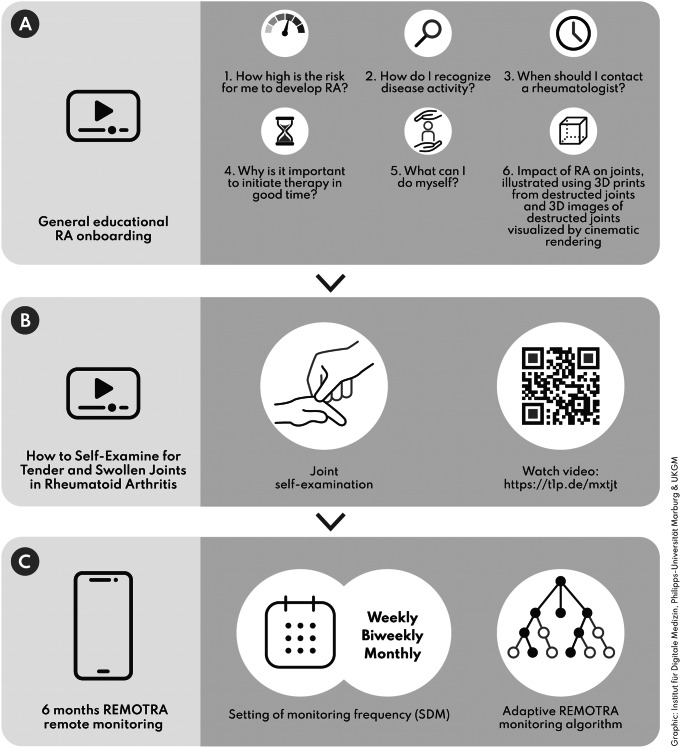
Overview of three digital self-monitoring program modules, including the (**A**) RA overview video (**B**) joint self-examination video and (**C**) REMOTRA remote monitoring decision support-system

The program included a (1) general RA educational video, a (2) joint self-examination video and (3) the REMOTRA remote monitoring decision support-system (Fig. 2).

Links to two standardized educational onboarding videos were sent to participants to foster patient empowerment and self-management. The first video (13 min) addressed five frequently asked questions of individuals at-risk for RA regarding the prodromal stage of RA and included 3-D joint images enhanced by cinematic rendering to illustrate potential damage of RA [20] (Fig. 2A). The video was created by Arnd Kleyer, who led the outpatient clinic for individuals at-risk for RA. The second video was an instruction video explaining how to perform a joint self-examination and the concept of the DAS28 score (14 min) [11]. The video was originally produced by patients and researchers, as part of the REMORA study [21] at the University of Manchester’s Center for the Epidemiology of Arthritis. The German version was created as part of the TELERA study [11].

Remote monitoring was based on the CE-certified monitoring software ABATON©. An adaptive electronic questionnaire-based algorithm was developed (Figure S1) based on previous experiences with the software in routine follow-up of RA patients and a remote monitoring trial of RA patients [11]. The questionnaire included typical RA symptoms, such as pain, swelling, morning stiffness and intake of pain medication. The questions were selected based on regular in-person visits and consideration of existing screening tools, such as the SPARRA questionnaire and the EULAR definition criteria [22–24]. Answers were weighted and added to a quantitative score from 0 to 15 points (Figure S1). A cut-off value from ≥ 10 was defined as potentially manifested RA. The questionnaire was designed adaptively, so that questionnaire burden was kept to a minimum for participants with no symptoms. Questionnaire frequency options were weekly, biweekly or monthly and a selection was made using shared-decision-making. A reminder system prompted participants for three consecutive days if they did not complete the questionnaires.

### Outcomes

Feasibility outcomes were quantitative and included acceptance, usability, monitoring adherence and diagnostic value. Acceptance and usability were assessed using electronic pseudonymized Google Forms questionnaires. Acceptance was assessed using the Net Promoter Score (NPS) [25]. The NPS is derived from a single question that asks participants how likely they are to recommend a product or service to a friend or patient. Participants respond using an 11-point scale, ranging from 0 (not at all likely) to 10 (extremely likely). Responses are categorized as follows: scores of 0 to 6 are considered detractors, 7 and 8 are classified as passives, and 9 to 10 are labeled as promoters. The NPS is then calculated by subtracting the percentage of detractors from the percentage of promoters. Video acceptance was queried directly after watching the videos. Monitoring acceptance using the NPS and usability of the monitoring software and algorithm using the System Usability Scale (SUS) [26] was assessed at month 3 and 6, The SUS score ranges from 0 (worst) to 100 (best), with scores above 68 considered above average and those above 80 regarded as high [27]. Additionally, SUS scores were translated into categories like “excellent” using the adjective rating scale, as previously described by Bangor et al. [28]. Monitoring adherence was defined as number of months in which the screening was fully completed within the specified interval. The diagnostic value of the REMOTRA algorithm to identify individuals with manifested RA was reported, stating sensitivity, specificity, positive predictive value and negative predictive value. Symptom burden was based on the questionnaire answer from participants. All calculations were performed using Microsoft Excel.

## Results

**Fig. 3:**
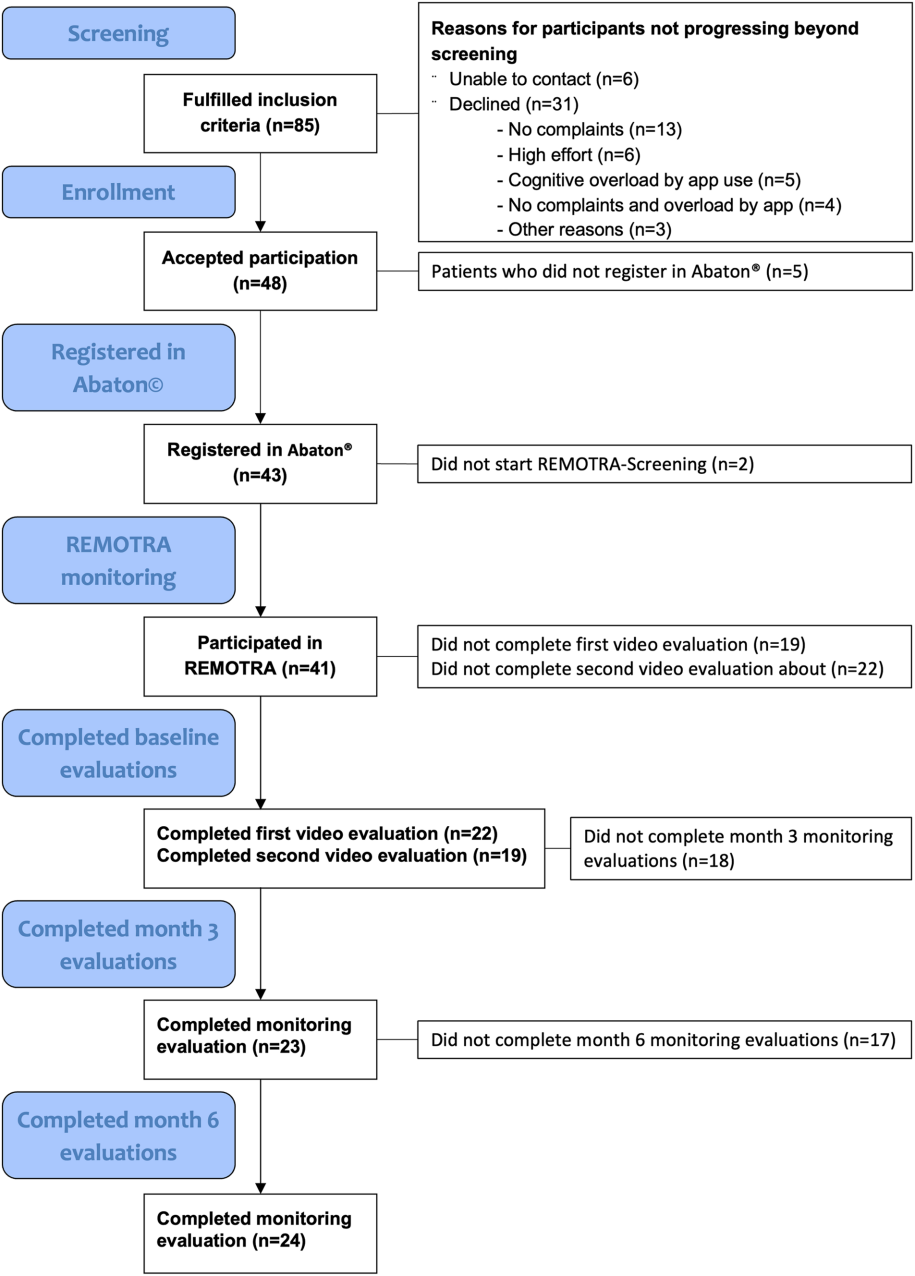
CONSORT flow diagram

A total of 85 participants were screened and 49 participants were recruited between November 8, 2021 and March 21, 2022. Reasons for non-enrolment are displayed in the CONSORT flow diagram (Fig. 3). Most common reason to decline was lack of disease symptoms. 43 participants started using the monitoring software and 41 patients completed at least one REMOTRA questionnaire. 27/41 (65.9%) participants were female. Mean age was 50.1 (SD: 13.7) years, 6/41 (14.6%) were active smokers. Median symptom duration was 48 months (IQR: 22–88 months).

Both videos were well accepted. The general RA education video had an NPS of 54.5, with 63.6% (*n* = 14/22) promoters, 9.1% (*n* = 2/22) detractors and 27.3% (*n* = 6/22) indifferent study participants. The self-examination video was rated with an NPS of 31.6, based on 57.9% (*n* = 11/19) promoters, 26.3% (*n* = 5/19) detractors and 15.8% (*n* = 3/19) indifferent participants.

3/41 (7.0%) study participants decided to answer the monitoring weekly, 14/41 (34.1%) biweekly and 24/41 (58.5%) monthly. 23/41 (56.1%) patients completed the evaluation after three months and 24/41 (58.5%) study participants after six months. Patient acceptance decreased from an NPS of 21.7 at three months to 8.3 at six months. At 3 months, 47.8% (*n* = 11/23) were promoters, 26.1% (*n* = 6/23) were detractors and 26.1% (*n* = 6/23) were indifferent, while at 6 moths 45.8% (*n* = 11/24) were promoters, 37.5% (*n* = 9/24) detractors and 16.7% (*n* = 4/24) indifferent. Software usability was continuously rated high with an average SUS of 88.1/100 (SD 5.5) at three months and 85.4/100 (SD 16.0) at six months. 24/41 patients (58.5%) completed each questionnaire at the specified interval. On average, the monitoring software was used regularly by participants for 4.8 months (SD: 1.8).

Overall, 25/41 (61.0%) of study participants had a score ≥ 10 at least once. All 3/41(7.3%) patients that were eventually diagnosed with RA during an in-person visit had a REMOTRA score ≥ 10. In contrast, 22/41 (53.7%) were not diagnosed with RA, although REMOTRA score was ≥ 10 points at least once. 16/41 (39.0%) of study participants always had a score off less than 10 points and none of these patients were diagnosed with RA. This data results in a sensitivity of 100% and a specificity of 42.1%. The positive predictive value lies at 12% and the negative predictive value is 100%.

Arthralgia was the leading reported symptom (32/41, 78.0%,), followed by joint swelling (30/41, 73.2%) and morning stiffness (18/41, 43.9%). 24/41 (58.5%) reported to have used analgesics and 12/41 (29.3%) used cortisone at least once. 7/41 (17.1%) patients reported to be completely symptom free. Fluctuation of symptoms occurred in 32/41 (86.5%) patients defined as changing REMOTRA scores.

## Discussion

This study is the first to explore remote digital monitoring during the at-risk phase of rheumatoid arthritis. The high levels of patient acceptance, usability, and adherence observed, along with promising diagnostic outcomes, emphasize the potential for routine clinical care.

Importantly, the study systematically uncovered the substantial symptom burden experienced by individuals at-risk for RA. Consistent with previous findings, joint pain was described as the most frequently reported symptom [23, 29]. Additionally, the unexpected observation that over half of the participants reported using analgesics and nearly one-third used corticosteroids shows that more attention should be paid to medication intake. Furthermore, it highlights the need for timely consideration of preventive immunosuppressive therapies, particularly in light of recent trials showing the efficacy of such interventions. For instance, using abatacept in the pre-clinical phase of RA did not only reduce inflammatory markers on MRI but also lowered the incidence of arthritis development as recently demonstrated in the ARIAA (Abatacept inhibits inflammation and onset of rheumatoid arthritis in individuals at high risk) trial [30].

The high negative predictive value of the REMOTRA algorithm suggests that its use could reduce the necessity for some in-person visits, thereby lowering healthcare costs. Furthermore, the reassuring feedback could reduce unnecessary patient anxiety. However, the low positive predictive value indicates that further refinement of the cut-off thresholds is required to enhance diagnostic accuracy. Incorporating additional objective data generated by patients at home [8], such as stepcount [31], smartphone camera-assisted joint analysis [32] and capillary self-sampling [33] could further improve diagnostic precision.

It is important to note that 31 participants (39.2%) declined to participate, primarily due to the absence of symptoms. This highlights a challenge commonly observed in remote monitoring studies, where symptom absence correlates with declining adherence, also in patients with established RA [21, 34]. Adherence in this study was good with 55.8% completing each questionnaire, whereby participants were either highly compliant or scarcely engaged with the monitoring. Prior research has shown that patients who have access to their digital monitoring data demonstrate better compliance compared to those who do not [35]. In this study, access to REMOTRA scores likely contributed to the observed adherence. Additionally, adherence could be further improved through enhanced patient education, which was provided in this study [36]. Further customization of the monitoring software could improve adherence [9, 34], as some participants suggested a daily documentation option. Future studies should consider more actively integrating monitoring results into clinical practice by discussing the data during in-person visits and potentially deferring visits when no concerning thresholds are reached. The objectives of the remote monitoring should also be specifically pointed out to individuals in the education videos.

The educational videos were generally well-received, and previous studies have highlighted the benefits of video-based education for RA patients [37, 38]. These benefits include improved patient self-management [39] and better compliance with treatment [40]. However, the limited availability of professionally produced educational videos poses a challenge, as patients may struggle to assess the quality of the content [41]. Therefore, physicians should recommend specific videos that they have reviewed or produced themselves [41].

Several limitations of this study should be acknowledged. The absence of blinding, randomization, may introduce bias. Despite the IRACE cohort being one of the largest for at-risk patients, the small number of participants who completed all evaluations limits the statistical power and generalizability of the findings. The six-month duration also constrained the number of patients who developed RA, as well as adherence and acceptance rates, which could decline in a longer study. Mandatory smartphone ownership introduced a selection bias and limited the generalizability of the findings. No direct patient involvement and feedback was considered at this stage to finalize the algorithm, however such co-development is planned to guide the next version and routine implementation. Additionally, incorporating qualitative methods such as interviews and focus groups could provide deeper insights into the reasons for non-participation and adherence challenges, thereby informing future improvements.

## Conclusion

This study highlights the feasibility and significant potential of a multimodal digital self-monitoring program for individuals at-risk for rheumatoid arthritis (RA). This approach could enhance continuity of care while enabling more informed and cost-efficient use of limited in-person visit slots. However, further refinement is necessary to boost patient uptake, adherence, and the diagnostic accuracy of REMOTRA. Additionally, further validation is crucial to fully translate the benefits of this approach into routine clinical practice.

## Electronic supplementary material

Below is the link to the electronic supplementary material.

Supplementary Material 1: The adaptive REMOTRA remote monitoring algorithm. Total number of questions vary based on symptom burden. A cut-off value of ≥ 10 pts was defined as manifested RA.

## Data Availability

The raw data supporting the conclusions of this article will be made available by the authors upon reasonable request.

## Abbreviations

ACPA: Anti-Citrullinated Peptide Antibodies
NPS: Net Promoter Score
RA: Rheumatoid Arthritis
RF: Rheumatoid Factor
SD: Standard Deviation
SUS: System Usability Scale
