# Supplementary figures and images for: Early detection of rheumatoid arthritis through patient empowerment by tailored digital monitoring and education: a feasibility study
Source: Rheumatol Int. 2025 Feb 4;45(2):43. doi: 10.1007/s00296-025-05793-8 (PMC11794354; doi:10.1007/s00296-025-05793-8)

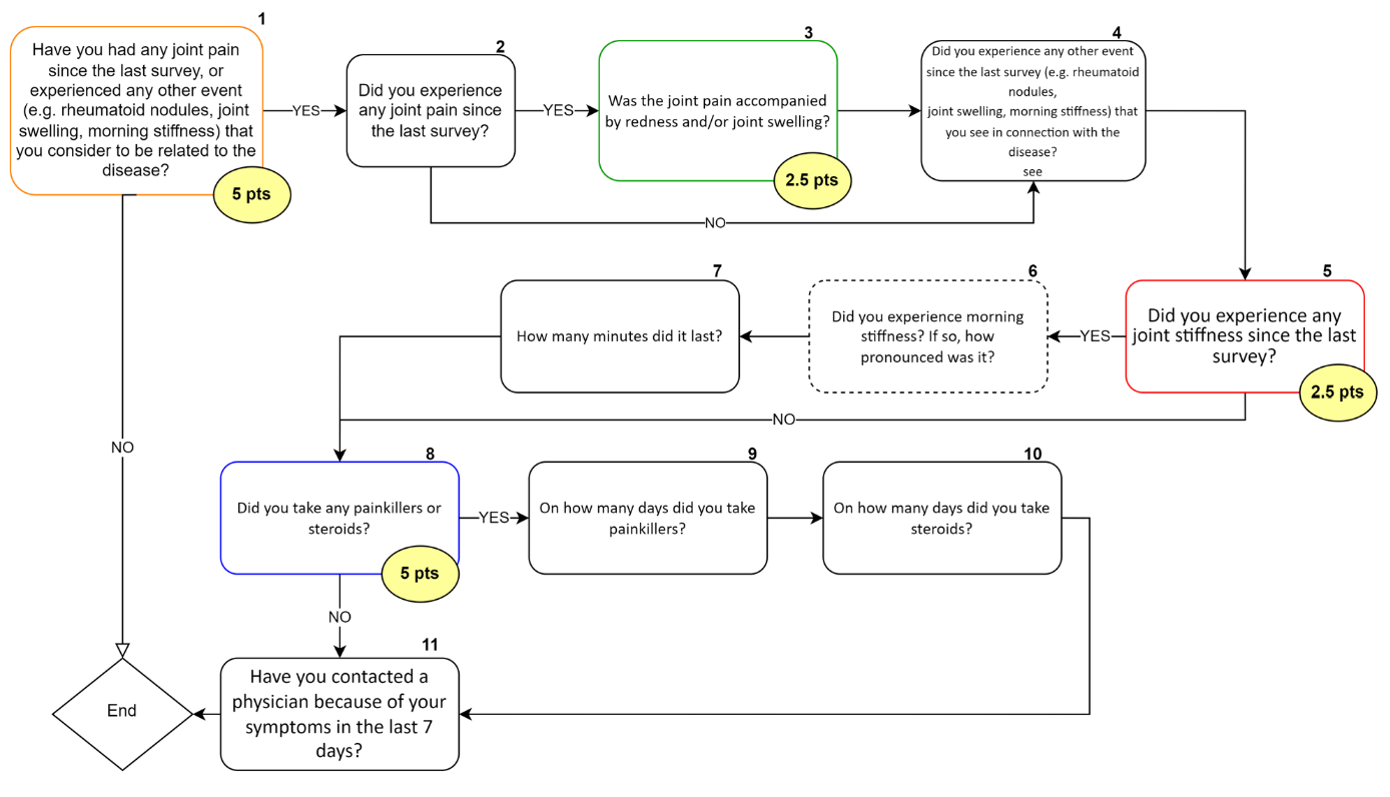

Supplement: Supplementary file 1 — Supplementary Material 1: The adaptive REMOTRA remote monitoring algorithm. Total number of questions vary based on symptom burden. A cut-off value of ≥ 10 pts was defined as manifested RA. [file 296_2025_5793_MOESM1_ESM.png]
